# Finite element analysis of long SI screws in the treatment of vertically unstable bilateral sacral fractures

**DOI:** 10.1371/journal.pone.0324612

**Published:** 2025-05-20

**Authors:** Wei Zhou, Xuan Pei, Jincheng Huang, Jianyin Lei, Ling Zhu, Guodong Wang, Junhong Lian, Ximing Liu

**Affiliations:** 1 Department of Orthopeadics Surgery, General Hospital of Central Theater Command of PLA, Wuhan, China; 2 Department of Spine Surgery, The Affiliated Hospital of Wuhan Sports University, Wuhan, China; 3 University Center of Orthopaedic, Trauma and Plastic Surgery, University Hospital Carl Gustav Carus at Technische Universität Dresden, Dresden, Germany; 4 Affiliated Second People’s Hospital, China Three Gorges University, Yichang, China; 5 Taiyuan University of Technology, Taiyuan, China; 6 Hubei University of Chinese Medicine, Wuhan, China; Iran University of Medical Sciences, IRAN, ISLAMIC REPUBLIC OF

## Abstract

**Objective:**

The finite element analysis (FEA) was used to investigate the biomechanical stability of combined long SI screws in the treatment of bilateral sacral fractures.

**Methods:**

This study was conducted between September 10, 2023, and June 13, 2024. Using the finite element (FE) method, a vertically unstable bilateral sacral fracture model and eight internal fixation groups were established: L1 (one long sacroiliac (SI) screw in S1), T1 (one transiliac-transsacral (TITS) screw in S1), 2S1 (two SI screws in S1 and S2), L1L2 (two long SI screws in S1 and S2), 2S1L2 (two SI screws in S1 and one long SI screw in S2), L1T2 (one long SI screw in S1 and one TITS screw in S2), T1T2 (two TITS screws in S1 and S2), and 2S1T2 (two SI screws in S1 and one TITS screw in S2). A pelvic standing posture was simulated to compare overall stiffness, average displacement along the fracture line, posterior rotation angle of the sacrum, and stress distribution in the SI joint among the models. In addition, stress nephograms of the eight internal fixations were analyzed.

**Results:**

Models with smaller sacral displacement generally exhibited greater stiffness. Two-segment fixation constructs (T1T2, L1T2, 2S1T2, L1L2) provided better biomechanical stability than single-segment fixations (T1, 2S1, L1). The L1T2 group demonstrated stiffness comparable to T1T2 and superior stability to 2S1T2 when two segments were fixed. Peak stress was highest in the L1 group (211.9 MPa) and lowest in the T1T2 group (107.1 MPa), with all models remaining within the safe range of titanium alloy. The stress on implants was mainly concentrated at the SI joint–screw interface. Two-segment fixations showed lower peak stress and more uniform stress distribution, suggesting better load-sharing and reduced implant fatigue risk. Sacral retroversion angles followed the same ranking as peak stress at the SI joint, with T1T2, L1T2, and 2S1T2 groups showing the smallest angles, indicating superior rotational stability.

**Conclusion:**

SI screws in dual-segment provide better biomechanical stability than those in the single-segment. Both the L1T2 and T1T2 groups demonstrate good biomechanical stability and are reliable for the fixation of vertically unstable bilateral sacral fractures. When a TITS screw cannot be inserted in the S1 segment, using a long SI screw in S1 combined with a TITS screw in S2 can achieve a comparable fixation effect.

## Introduction

Vertically unstable sacral fractures belong to the category of Tile C-type pelvic fractures, which are often caused by high-energy traumatic events, such as crush injuries or falls from a height. When sacral fractures are associated with significant longitudinal displacement, they are frequently accompanied by soft tissue injuries, leading to severe and highly unstable posterior pelvic ring injuries [1,2]. Conservative management of vertically unstable sacral fractures is often associated with suboptimal outcomes and a high risk of complications, including fracture nonunion, sacral malunion, chronic low back pain, and limb length discrepancy. Therefore, surgical intervention is commonly recommended in clinical practice [3]. Following closed reduction, percutaneous SI screw placement has become a commonly employed technique for the treatment of sacral fractures. However, due to the significant longitudinal shear forces at the fracture site, SI screw fixation may lead to complications such as loosening, breakage, and failure [4,5]. In recent years, SI screws have evolved from traditional lengths to long SI screws and TITS screws, enhancing the stability of the SI joint [6,7]. Owing to their superior stability, long SI screws and TITS screws have increasingly been utilized in the treatment of vertically unstable bilateral sacral fractures. The choice of fixation segment-S1, S2, or both-is typically guided by the fracture morphology and bone quality. However, despite their growing clinical application, there is still a lack of biomechanical studies directly comparing various SI screw types and combinations in the treatment of vertically unstable bilateral sacral fractures. Further research is needed to determine which fixation strategies offer optimal biomechanical stability in such cases.

In this study, FEA was used to construct a model of the pelvis with vertically unstable bilateral sacral fractures. The internal fixation models were created based on commonly used combinations of SI screws in clinical practice. This study aims to provide a theoretical foundation for selecting the most appropriate internal fixation combinations in the treatment of vertically unstable bilateral sacral fractures by analyzing and comparing the biomechanical stability of these fixation models.

## Materials and methods

### Ethics statement

This study was carried out in accordance with the Code of Ethics of the World Medical Association (Declaration of Helsinki) and approved by the Ethics Committee of General Hospital of Central Theater Command of PLA (ref. no. 2020–027). A computed tomography (CT) of the pelvis was performed after informed consent of the volunteers, and the relevant data were authorized for academic exchange.

### Computational model

**Fig 1:**
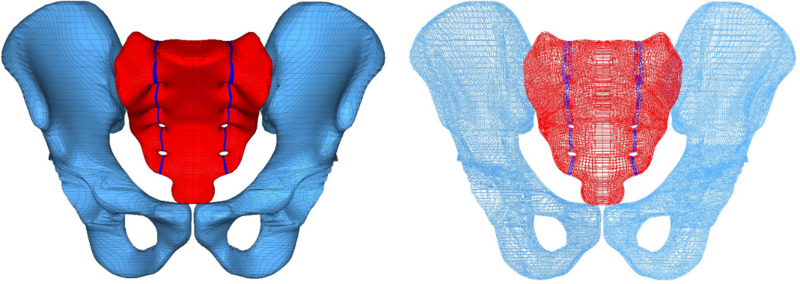
Completed reduction of vertical unstable bilateral sacral fractures.

**Fig 2:**
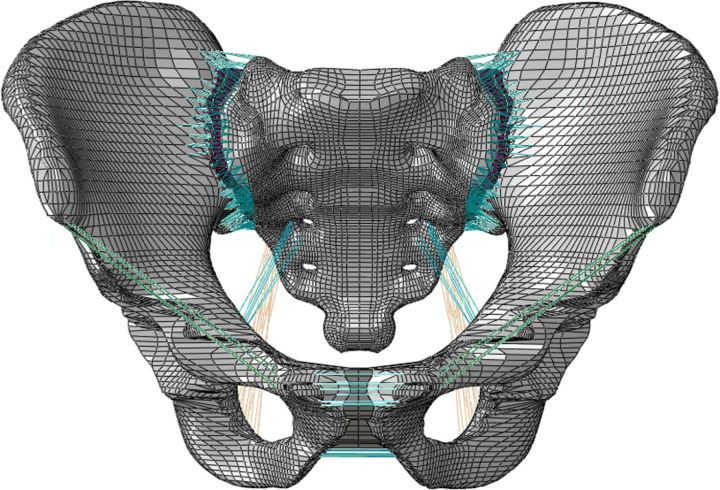
Normal pelvic FE model.

**Fig 3:**
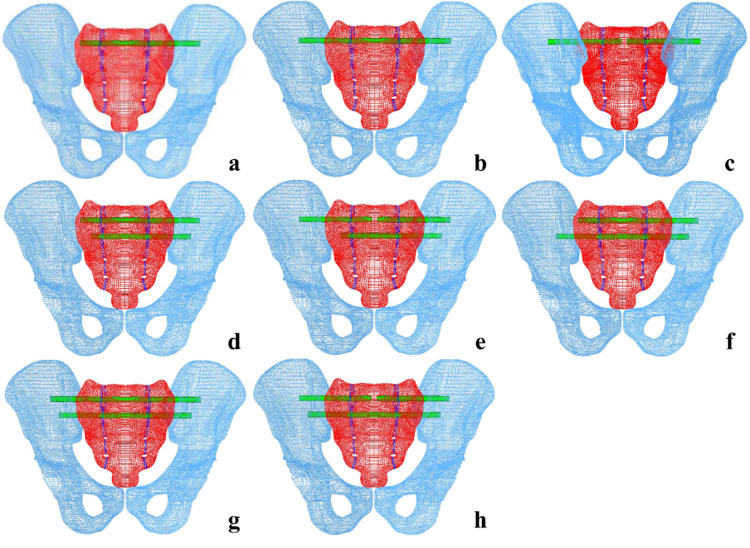
Illustration of eight internal fixation methods for vertically unstable bilateral sacral fracture. (a) one long SI screw in S1. (b) one TITS screw in S1. (c) two standard SI screws in S1 and S2. (d) two long SI screws in S1 and S2. (e) two SI screws in S1 and one long SI screw in S2. (f) one long SI screw in S1 and one TITS screw in S2. (g) two TITS screws in S1 and S2. (h) two SI screws in S1 and one TITS screw in S2.

A healthy adult volunteer (male, 31 years old, 178 cm, 75 kg) was selected for the study. The geometric model was created by importing DICOM images into Mimics 21.0 (Materialize, Inc., Leuven, Belgium), followed by editing and optimization using Geomagic Studio 10.0 (Geomagic Inc., USA). The 3D model was segmented with Solidworks 2017 (Dassault, France), to create a bilateral sacral fracture model. The vertical fracture line is located at the center of the sacral foramina on both sides (Fig 1). The elastic modulus of the fracture lines was set to one-tenth of the normal bone, with bilateral fracture gap displacement controlled to under 1 mm. S1 and S2 SI screws, long SI screws, and TITS screws were designed in Hypermesh 14.0 (Altair Inc., USA) under the guidance of medical professionals and equipment engineers. The complete FE model was then meshed in the same software (Fig 2). C3D6 elements were chosen to model the bones and the instrumentation. The models included L1 (one long SI screw in S1), T1 (one TITS screw in S1), 2S1 (two screws in S1 and S2), L1L2 (two long screws in S1 and S2), 2S1L2 (two screws in S1, one long screw in S2), L1T2 (one long screw in S1, one TITS screw in S2), T1T2 (two TITS screws in S1 and S2), and 2S1T2 (two screws in S1, one TITS screw in S2) (Fig 3). All screws were modeled with a 6.5 mm diameter (Watson Corporation, Changzhou, China). The screw design was simplified to a cylindrical shape for modeling purposes. The meshed model was imported into Abaqus 2020 software, and material properties were assigned [8] (Table 1). The material properties were defined as titanium alloy, with an elastic modulus of 110 GPa and a Poisson’s ratio of 0.3. The cortical bone of the sacrum and iliac bone were modeled as 1.5 mm thick shell [9,10]. Ligaments were delineated at corresponding nodes on the model’s surface based on anatomical locations and simulated using T3D2 elements [9] (Table 2). The simulated ligaments included the pubic ligament, SI ligament, inguinal ligament, superior pubic ligament, sacrospinous ligament, and sacrotuberous ligament [11,12]. The full pelvic FE model is shown in Fig 2. The FE model of the entire pelvis established in this experiment consisted of 112,002 elements and 115,940 nodes.

**Table 1:** The material properties used in the FE models.

Material	Elastic modulus (MPa)	Poisson^,^s ratio	Number of units
Sacral cartilage	1000	0.30	470*2
Femoral Cartilage	1000	0.30	1772*2
Iliac endplate	500	0.25	465*2
Iliac cartilage	1000	0.30	468*2
Sacral endplate	500	0.25	2493*2
Cortical bone	17,000	0.30	5,5792
Cancellous bone	150	0.20	6,5536

**Table 2:** The material properties of main ligaments in models.

Material of ligament	Stiffness coefficient (N/mm)	Number of units
SI ligament	5000	161*2
Sacrospinous ligament	1500	161*2
Sacrotuberous ligament	1500	566*2
Suprapubic ligament	500	123
Arcuate pubic ligament	500	187
Inguinal ligament	250	191*2

For S1 SI screws, the entry point is located at the posterior 1/3 along the line connecting the anterior and posterior superior iliac spines. The long SI screw extends to the contralateral sacral wing (Fig 3a), the TITS screw penetrates the contralateral ilium (Fig 3b) and the tip of the SI screw reaches the midline of the sacrum (Fig 3c). For S2 SI screws, the entry point is located at the posterior 1/5 of the line connecting the sacral promontory and posterior superior iliac spine. The screws traverse the SI joint and extend into the midline of the S2 vertebral body. The screw lengths were set at 80 mm for standard SI screws, 127 mm for long SI screws, and 170 mm for TITS screws.

### Finite element model validation

FE models were constructed according to methods described in previous studies [13]. The results were validated using in reported in cadaveric [14] and in vitro data [12,13]. Five translational loads (294 N) and three rotational moments (42 N*m) (anterior, posterior, superior, inferior, mediolateral, flexion, extension, and axial rotation) were tested.

### Loading and boundary condition

A standing posture was simulated by constraining the distal ends of both femurs and the six directions of the left and right ilium, ischial tubercle surface, and acetabulum. In reference to previous studies, a vertical force of 600 N [15] was applied to the top surface of S1 to simulate body weight. Binding constraints were applied between the ilium, sacrum, and SI joints. Surface-to-surface contact was defined between cartilage interfaces and fracture surfaces. Additionally, binding constraints were applied between the screws and surrounding bone. All simulations were performed using Abaqus 2020 software.

### Evaluation index

Maximum sacral displacement and model stiffness, average displacement along the fracture line, peak stress and stress distribution in the implant, peak stress in the SI joint and posterior rotation angle of the sacrum were calculated under the standing loading conditions. A smaller sacral displacement reflects better posterior pelvic ring stability, making it a key indicator of fixation effectiveness. Model stiffness, which represents the ability of the structure to resist elastic deformation under load, provides a complementary measure of stability [11]. To further evaluate fixation stability at the fracture site, twenty nodes along the fracture line were selected to calculate the average displacement for each model. Higher peak stress and more concentrated stress distribution suggest increased load transmission and a higher risk of implant failure [12]. In addition, elevated peak stress in the SI joint was recorded, as excessive stress in this region can contribute to fixation-related complications such as joint degeneration or screw loosening. Finally, the posterior rotation angle of the sacrum under sagittal loading was measured to assess rotational stability of the posterior pelvic ring. A larger rotation angle reflects inferior rotational control, indicating reduced fixation stability.

## Results

### Validation of the pelvic model

The FE models were constructed based on methods described in previous studies [13]. Model validation was performed using cadaveric data [14] and in vitro experimental results [13]. The FE model was tested under five translational loads (294 N) and three rotational moments (42 N·m), including anterior, posterior, superior, inferior, mediolateral translation, as well as flexion, extension, and axial rotation. In comparison with the Miller model, all test data fell within the standard error range, demonstrating good overall agreement with the previously published results [16].

### The maximum sacral displacement, model stiffness and the average displacement at the fracture line

**Fig 4:**
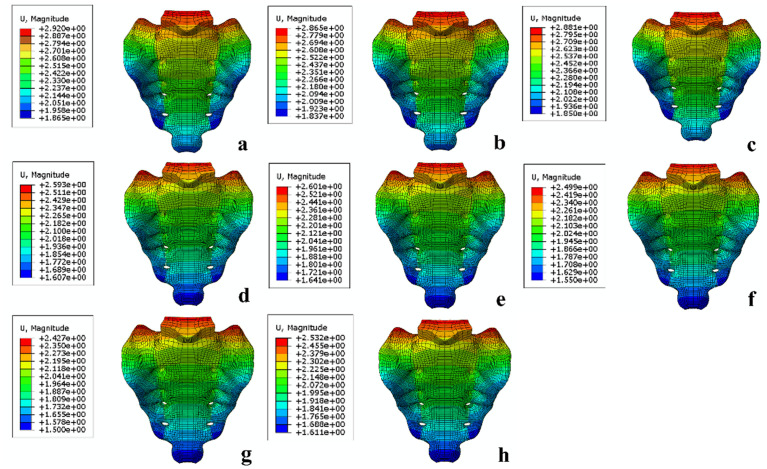
The sacral displacement magnitude in each model. (a) L1. (b) T1. (c) 2S1. (d) L1L2. (e) 2S1L2 (f) L1T2. (g) T1T2. (h) 2S1T2.

The maximum sacral displacement in each model, from largest to smallest, was: L1 > 2S1 > T1 > 2S1L2 > L1L2 > 2S1T2 > L1T2 > T1T2 (Fig 4). In contrast, the overall stiffness ranked as: T1T2 > L1T2 > 2S1T2 > L1L2 > 2S1L2 > T1 > 2S1 > L1. These results show that models with smaller displacement generally had greater stiffness. After internal fixation, all models showed improved stiffness. Two-segment fixation constructs that employed TITS or long SI screws, as seen in the T1T2, L1T2, and L1L2 groups, exhibited superior biomechanical performance compared to single-segment fixation configurations such as T1, 2S1, and L1. Under two-segment fixation, the L1T2 group exhibited stiffness similar to that of the T1T2 group and provided greater stability than the 2S1T2 group. The ranking of average displacement along the fracture line was consistent with sacral displacement: L1 > 2S1 > T1 > 2S1L2 > L1L2 > 2S1T2 > L1T2 > T1T2. Displacement distribution along the path in the T1T2, L1T2, and 2S1T2 groups overlapped more, indicating fixation stability. In comparison, the L1L2 and 2S1L2 groups showed moderate overlap, while T1, 2S1, and L1 had greater variability, suggesting less stable fixation.

### The peak stress and stress distribution in the internal fixation models

**Fig 5:**
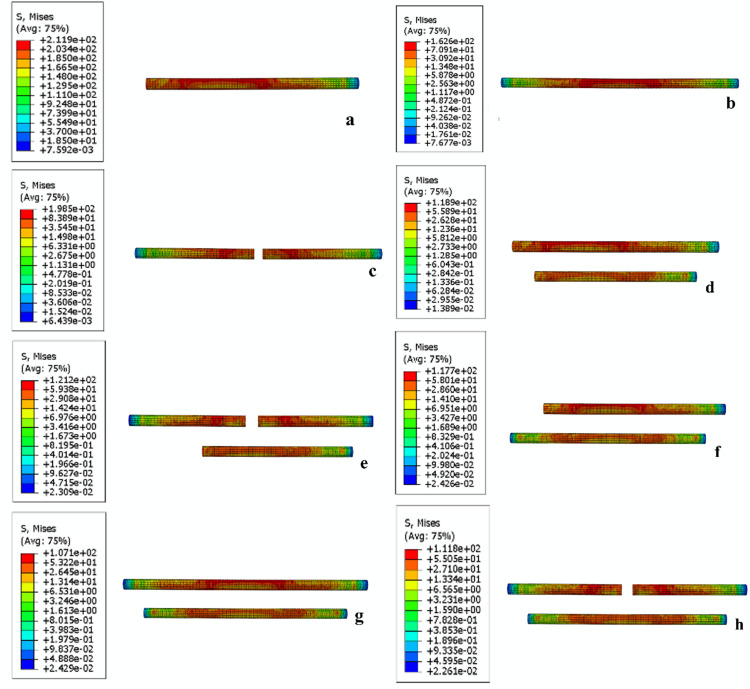
The stress distribution in each model. (a) L1. (b) T1. (c) 2S1. (d) L1L2. (e) 2S1L2 (f) L1T2. (g) T1T2. (h) 2S1T2.

The peak stress values in the internal fixation models, ranked from highest to lowest, were as follows: L1 > 2S1 > T1 > 2S1L2 > L1L2 > 2S1T2 > L1T2 > T1T2 (Fig 5). The L1 group exhibited the highest peak stress at 211.9 MPa, whereas the T1T2, 2S1T2, and L1T2 groups demonstrated substantially lower values of 107.1 MPa, 111.8 MPa, and 117.7 MPa, respectively (Table 3). All peak stress values were well below the yield strength of titanium alloy, indicating the mechanical safety of all fixation groups. Stress concentrations were primarily located at both sides of the fracture lines, where the mechanical load is transferred between bone and implant. Notably, the T1T2, 2S1T2, and L1T2 groups not only exhibited lower peak stress values but also demonstrated a more uniform stress distribution across the internal fixation groups compared to the other models. This suggests improved load-sharing capacity and reduced risk of implant fatigue in these models.

**Table 3:** FEA results of various internal fixation models.

Groups	Sacral Displacement (mm)	Overall stiffness(N/mm)	Average displacement at the fracture line (mm)	Peak stress in the internal fixation (MPa)	Peak stress values at the SI joint (MPa)	Sacral retroversion angle (°)
**L1**	2.920	205.48	2.289	211.9	7.513	0.823
**2**S1	2.881	208.26	2.281	198.5	7.305	0.785
**T1**	2.865	209.42	2.272	162.6	7.125	0.776
**2**S1**L2**	2.626	228.48	2.087	121.2	6.481	0.591
**L1L2**	2.593	231.39	2.037	118.9	6.218	0.602
**2**S1**T2**	2.532	236.97	2.010	111.8	6.116	0.575
**L1T2**	2.499	240.10	1.962	117.7	5.829	0.582
**T1T2**	2.427	247.22	1.906	107.1	5.569	0.573

### The peak stress values at the SI joint and the sacral retroversion angle

**Fig 6:**
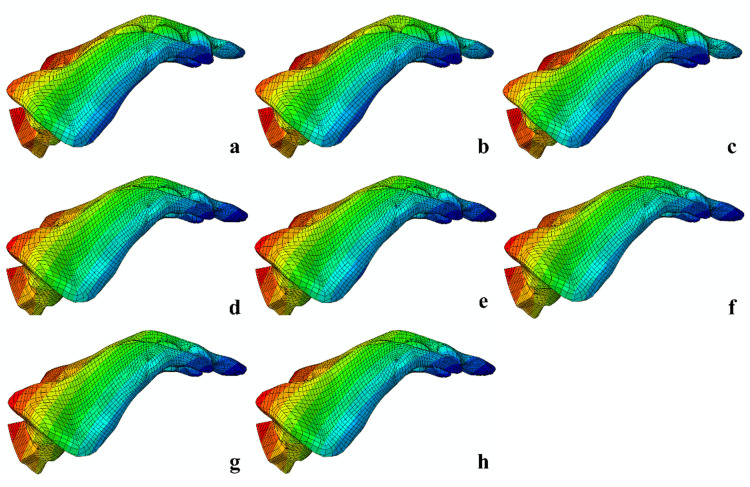
The sacral retroversion angles in each model. (a) L1. (b) T1. (c) 2S1. (d) L1L2. (e) 2S1L2 (f) L1T2. (g) T1T2. (h) 2S1T2.

The peak stress values at the bilateral SI joints, ranked from highest to lowest, were as follows: L1 > 2S1 > T1 > 2S1L2 > L1L2 > 2S1T2 > L1T2 > T1T2. Stress was primarily concentrated at the intersection of the SI joint and the screw. Using the center point of the S1 vertebral body endplate and the coccyx center as reference landmarks, sacral rotational displacement was measured and converted into sacral retroversion angles. The resulting retroversion angles, from highest to lowest, followed the same trend: L1 > 2S1 > T1 > 2S1L2 > L1L2 > 2S1T2 > L1T2 > T1T2 (Fig 6). The T1, L1, and 2S1 groups exhibited the largest sacral retroversion angles, indicating poor rotational control. In contrast, the L1L2 and 2S1L2 groups showed notable reductions in retroversion angles. Among all groups, the T1T2, 2S1T2, and L1T2 groups demonstrated the smallest sacral retroversion angles, reflecting improved rotational stability.

## Discussion

Vertically unstable sacral fractures are typically caused by high-energy trauma and lead to varying degrees of posterior pelvic ring instability, thereby necessitating individualized fixation strategies [17,18]. Percutaneous SI screw fixation is widely used due to its minimally invasive approach and reliable fixation strength [1,3,6,17]. However, given the substantial longitudinal shear forces in these fractures, standard SI screws are prone to complications such as loosening, breakage, and fixation failure [19].

Biomechanical studies have demonstrated that shear forces are aligned with the long axis of the screw, and that longer screws offer improved stress distribution, reduced stress concentration, and enhanced resistance to displacement [12]. Compared to standard SI screws, long SI screws offer wider clinical indications and better mechanical performance, while also demonstrating lower complication rates, such as screw breakage [20]. Additionally, TITS screws traverse both SI joints and engage bilateral iliac cortical bone, providing enhanced fixation strength and more uniform stress distribution. However, the efficacy of these screws relies on precise placement, which can be challenging in the presence of anatomical variations such as sacral dysmorphism [21].

Although SI screw fixation has been extensively studied in unilateral sacral fractures, there is a paucity of research focusing on bilateral fractures, especially those employing combined long SI screw [13,14,22,23]. To address this gap, we conducted a FEA to evaluate eight clinically applicable fixation strategies for vertically unstable sacral fractures. Among all models, the T1T2, L1T2, and 2S1T2 groups consistently exhibited superior biomechanical performance. Both the T1T2 and L1T2 groups demonstrated comparable reductions in sacral displacement and fracture line displacement, indicating robust fixation stability. Notably, the L1T2 group serves as a practical solution in cases where TITS screw placement at S1 is unfeasible due to sacral dysplasia or lumbosacral transitional vertebrae, conditions frequently encountered in clinical settings [24]. Analysis of the stress distribution on the screws further supports these findings.

The L1T2 and T1T2 groups exhibited the lowest peak stress values in both the implants and SI joints, indicating reduced risk of fixation fatigue or failure. Clinical studies have reported a higher risk of internal fixation failure, including screw loosening and breakage, especially when using standard SI screws for vertically unstable sacral fractures [20,25]. In particular, previous clinical reports have highlighted a notable incidence of screw breakage in cases of bilateral sacral fractures treated with standard SI screw fixation [26]. Our FEA supports these clinical findings and further reveals that the L1 and 2S1 groups, both utilizing standard or SI screws without dual-segment fixation, exhibited the highest peak stresses and greatest displacements. In contrast, the L1T2 and T1T2 groups significantly reduced stress concentrations and sacral retroversion angles, indicating superior mechanical performance and a decreased risk of implant-related complications. The stresses on implants were well below the yield strength of titanium alloy, indicating that the fixation groups are biomechanically safe under the applied loading conditions.

Despite these promising results, several assumptions and simplifications in our FE modeling should be acknowledged as limitations. First, the pelvis was modeled from a single healthy adult male CT scan, which may not capture anatomical variations found in elderly or osteoporotic patients. Second, the screw-bone interface was simplified using ideal bonded constraints, without simulating potential micro-movements or loosening at the bone-implant interface. Third, the screws were modeled as smooth cylinders, omitting the thread geometry, which may influence local stress distribution. Fourth, only static axial loading simulating a standing posture (600 N) was applied, whereas in vivo loading includes dynamic and multidirectional forces. Finally, although ligaments were incorporated, muscular forces and their stabilizing effects were not included in the model.

## Conclusions

In summary, dual-segment SI screw fixation provides superior biomechanical stability compared to single-segment fixation. Both configurations involving two TITS screws and those combining one long SI screw with one TITS screw demonstrated enhanced mechanical performance. These approaches appear to be safer and more reliable for stabilizing vertically unstable bilateral sacral fractures. Furthermore, in cases where TITS screw placement is not possible in the S1 segment, using a long SI screw in the S1 segment, in combination with TITS screw fixation in the S2 segment, can achieve effective stabilization.

## Supporting information

S1 Appendix Overall stiffness (Nmm) in eight models. (ZIP)

S2 Appendix Average displacement at the fracture line (mm) in eight models. (ZIP)

S3 Appendix Peak stress values at the SI joint (MPa) in eight models. (ZIP)

S4 Appendix Sacral retroversion angle (°) in eight models. (ZIP)
