# Supplementary figures and images for: Finite element analysis of long SI screws in the treatment of vertically unstable bilateral sacral fractures
Source: PLoS One. 2025 May 20;20(5):e0324612. doi: 10.1371/journal.pone.0324612 (PMC12091830; doi:10.1371/journal.pone.0324612)

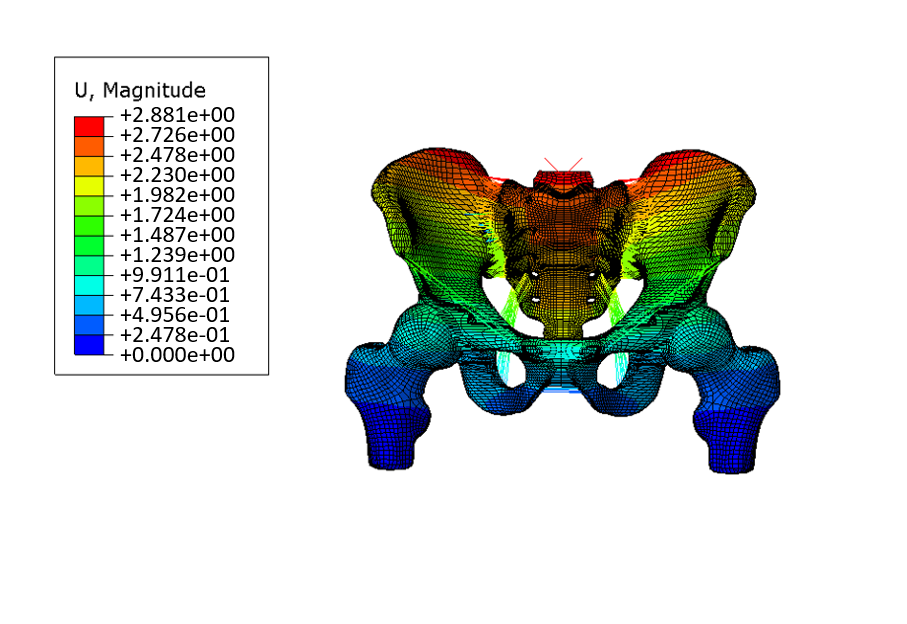

Supplement: S1 Appendix — (ZIP) [file pone.0324612.s001.zip › S1 Appendix/2S1.png]

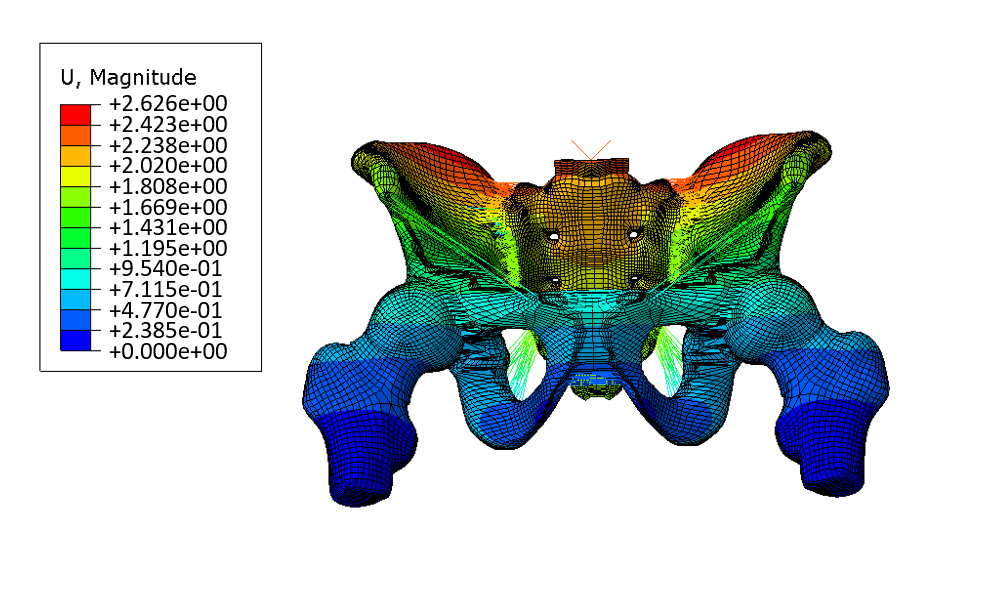

Supplement: S1 Appendix — (ZIP) [file pone.0324612.s001.zip › S1 Appendix/2S1L2.png]

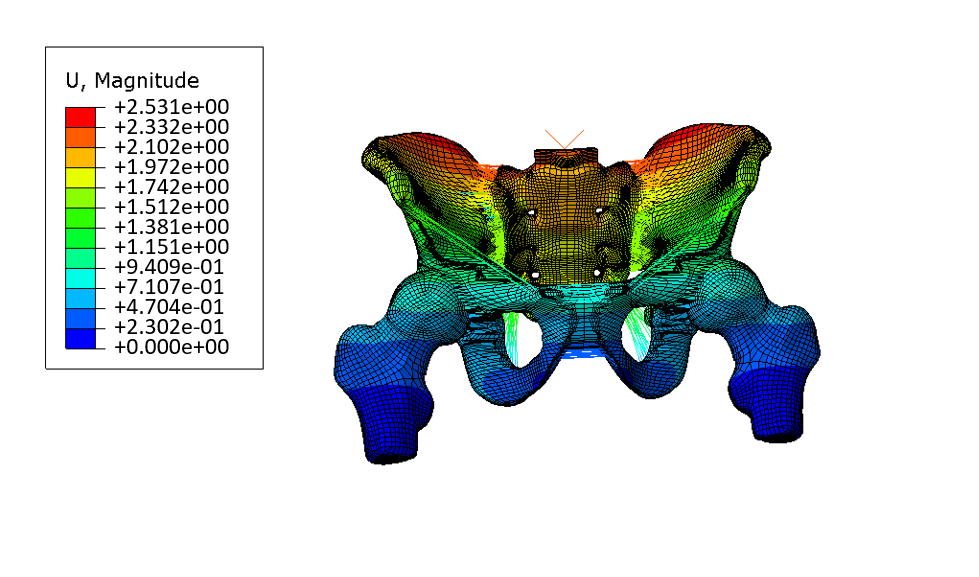

Supplement: S1 Appendix — (ZIP) [file pone.0324612.s001.zip › S1 Appendix/2S1T2.png]

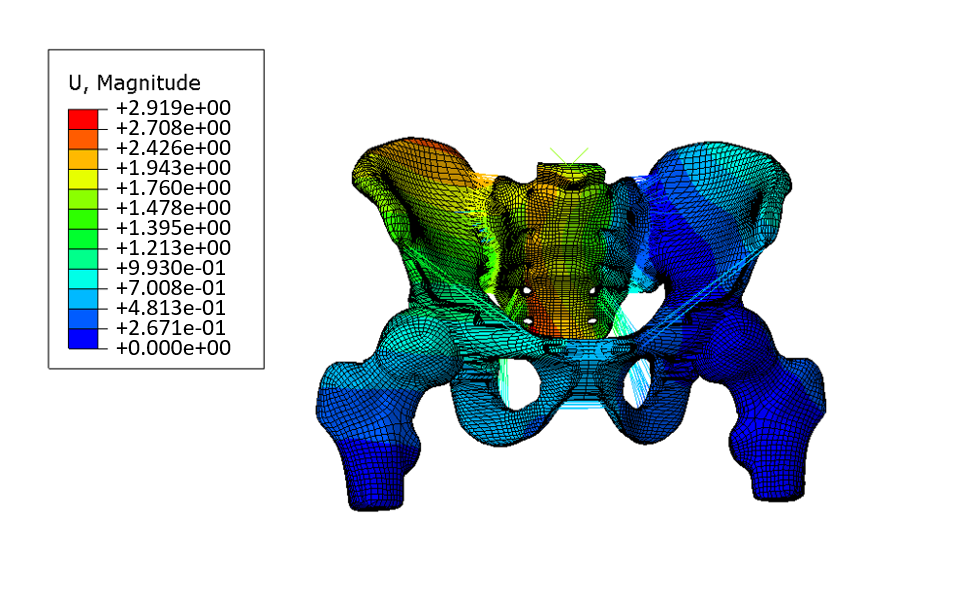

Supplement: S1 Appendix — (ZIP) [file pone.0324612.s001.zip › S1 Appendix/L1.png]

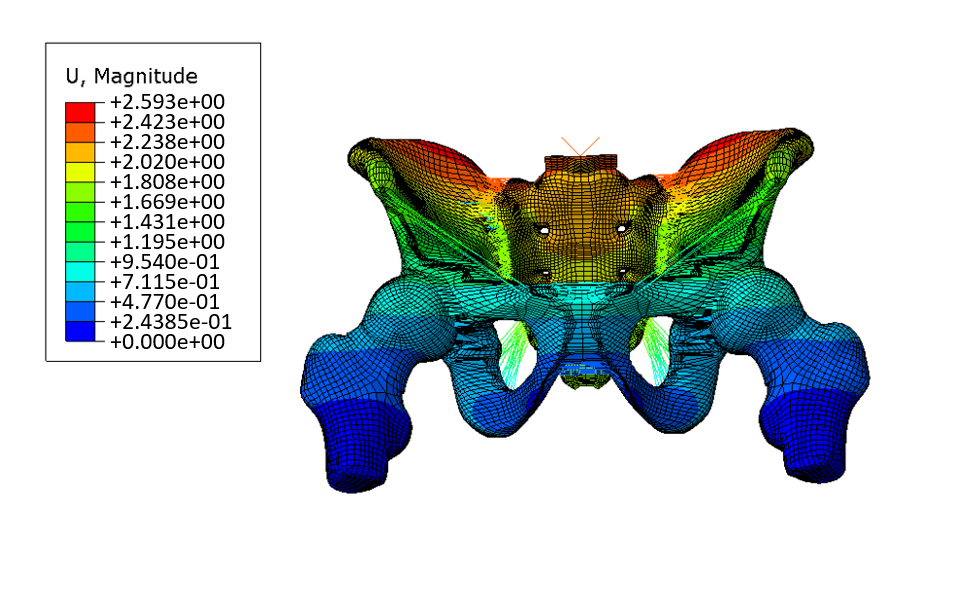

Supplement: S1 Appendix — (ZIP) [file pone.0324612.s001.zip › S1 Appendix/L1L2.png]

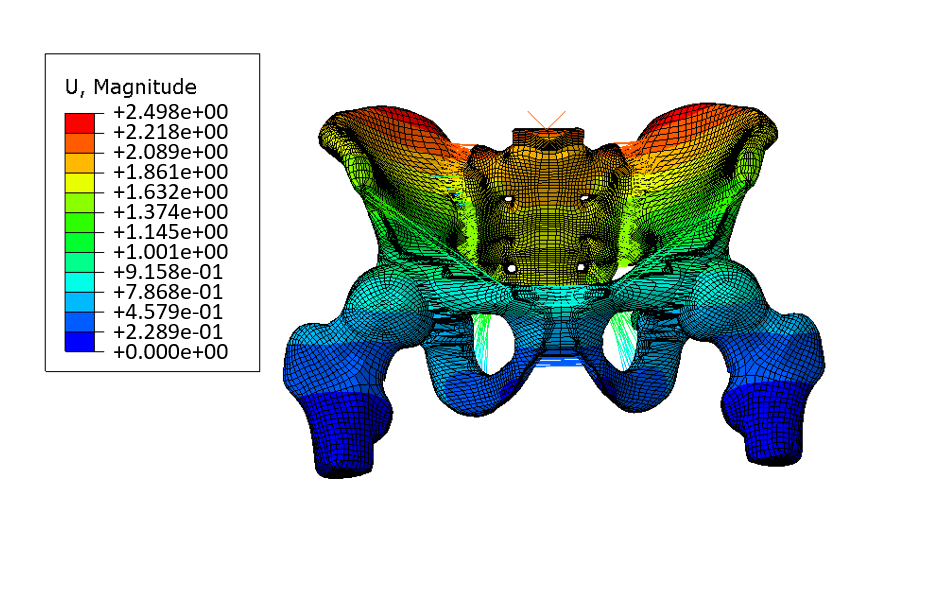

Supplement: S1 Appendix — (ZIP) [file pone.0324612.s001.zip › S1 Appendix/LIT1.png]

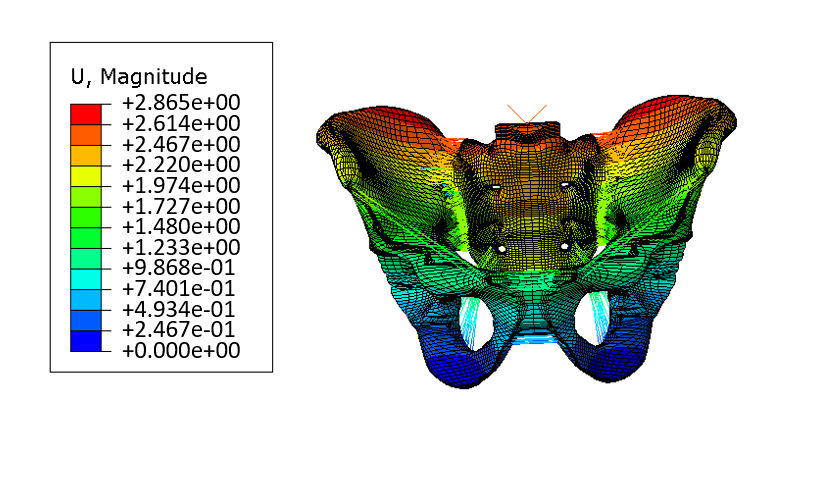

Supplement: S1 Appendix — (ZIP) [file pone.0324612.s001.zip › S1 Appendix/T1.png]

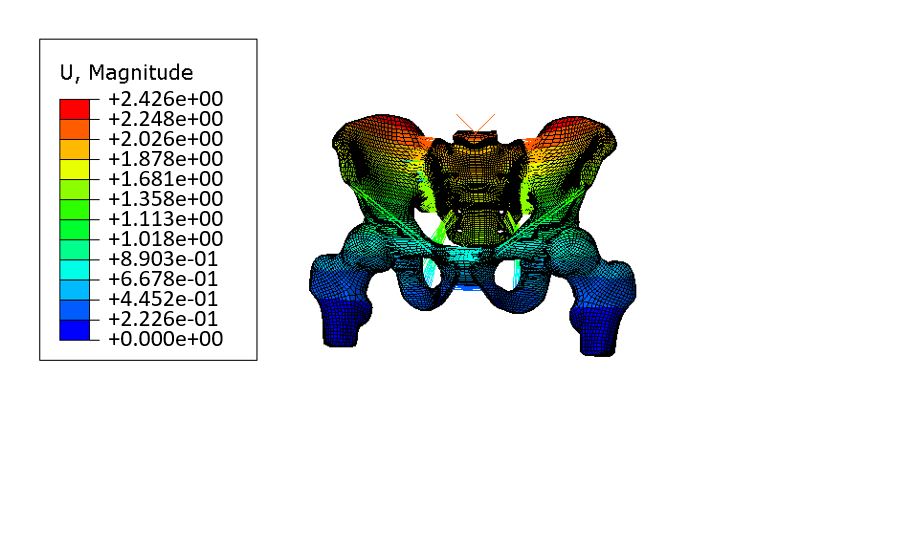

Supplement: S1 Appendix — (ZIP) [file pone.0324612.s001.zip › S1 Appendix/T1T2.png]

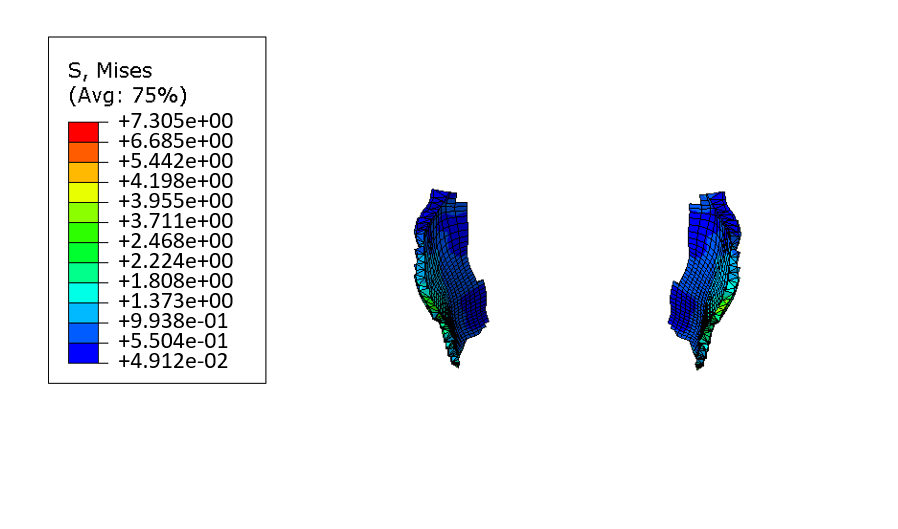

Supplement: S3 Appendix — (ZIP) [file pone.0324612.s003.zip › S3 Appendix/2S1.png]

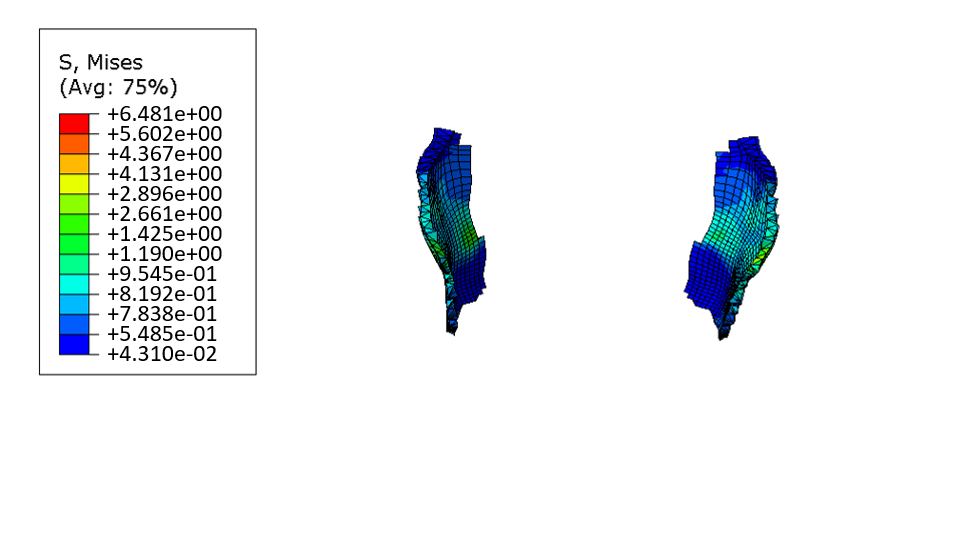

Supplement: S3 Appendix — (ZIP) [file pone.0324612.s003.zip › S3 Appendix/2S1L2.png]

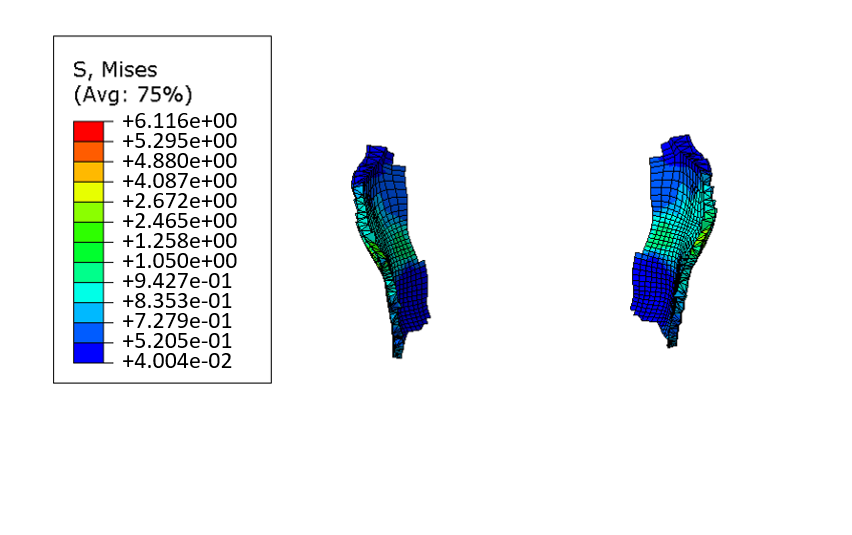

Supplement: S3 Appendix — (ZIP) [file pone.0324612.s003.zip › S3 Appendix/2S1T2.png]

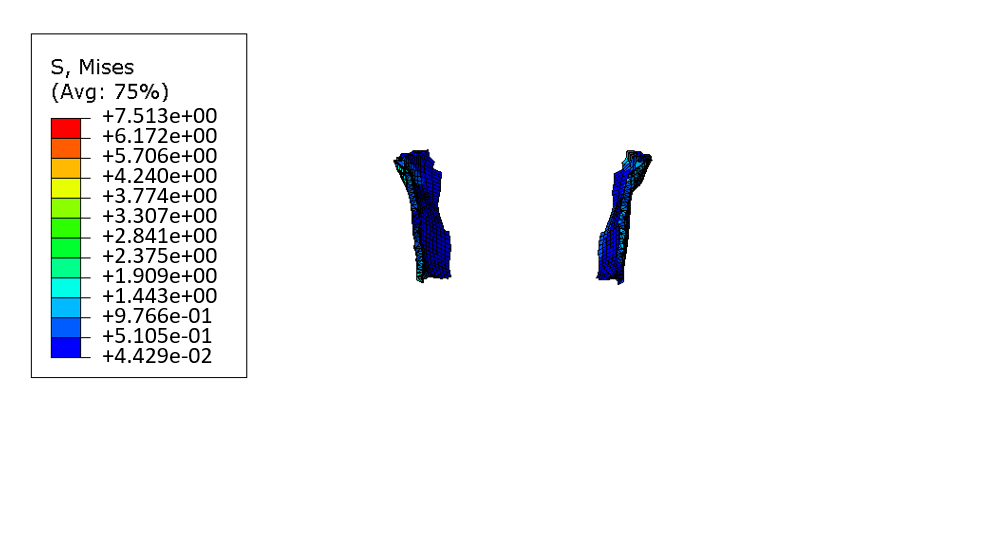

Supplement: S3 Appendix — (ZIP) [file pone.0324612.s003.zip › S3 Appendix/L1.png]

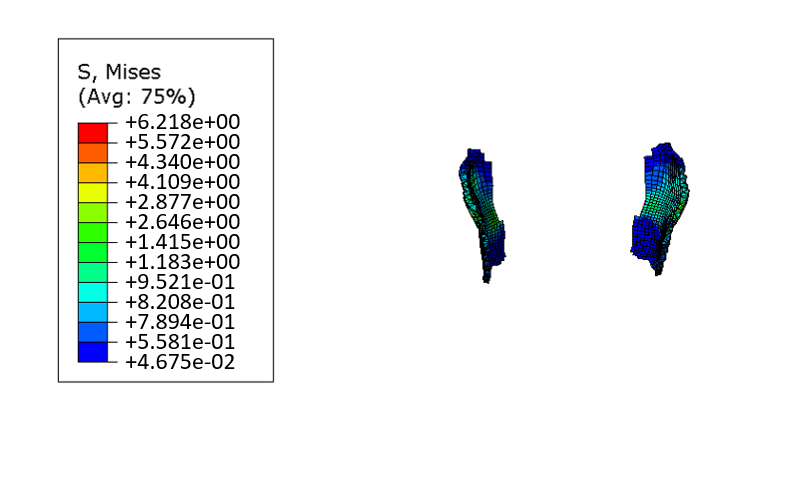

Supplement: S3 Appendix — (ZIP) [file pone.0324612.s003.zip › S3 Appendix/L1L2.png]

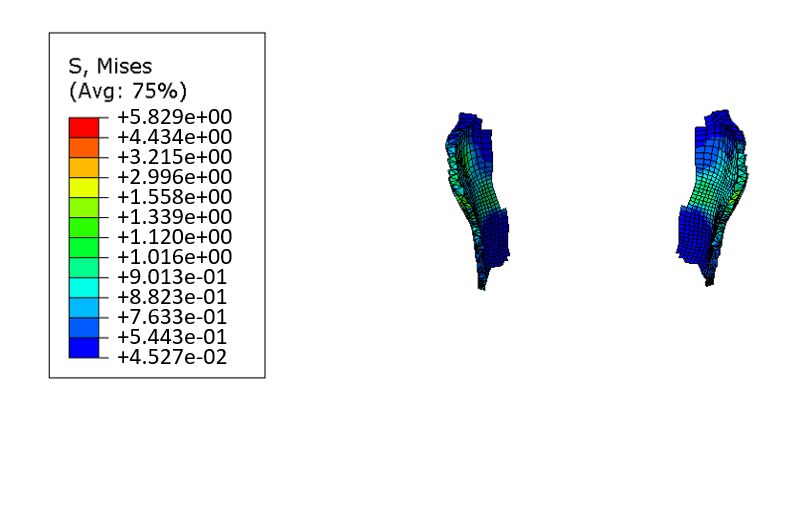

Supplement: S3 Appendix — (ZIP) [file pone.0324612.s003.zip › S3 Appendix/L1T2.png]

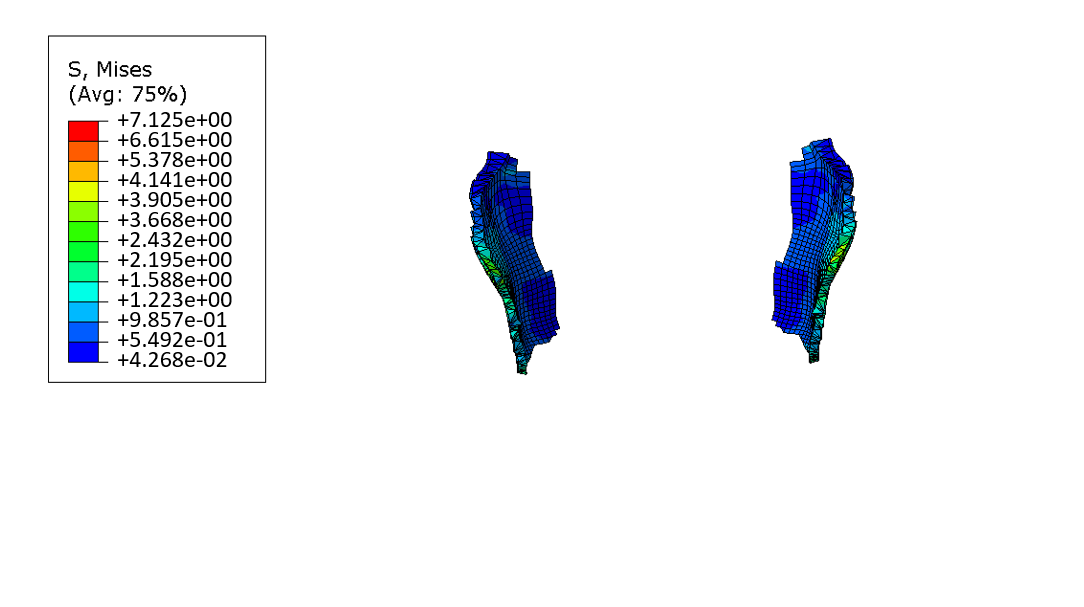

Supplement: S3 Appendix — (ZIP) [file pone.0324612.s003.zip › S3 Appendix/T1.png]

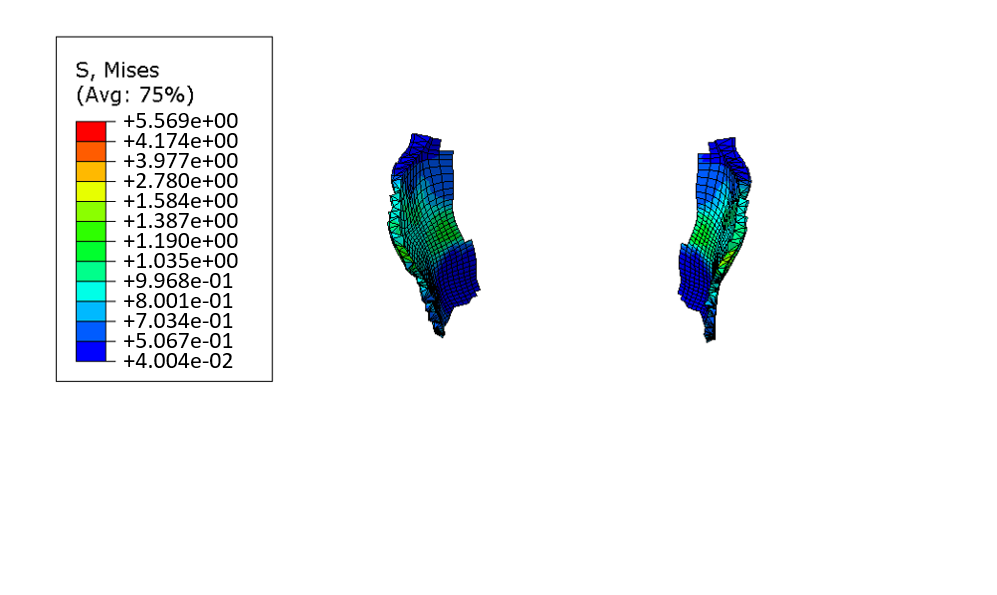

Supplement: S3 Appendix — (ZIP) [file pone.0324612.s003.zip › S3 Appendix/T1T2.png]
